# Di-μ_2_-cyanido-dicyanidobis{2,2′-[ethane-1,2-diylbis(nitrilo­methyl­idyne)]diphenolato}(1,4,8,11-tetra­azacyclo­tetra­deca­ne)dichromium(III)nickel(II) methanol disolvate

**DOI:** 10.1107/S1600536810016995

**Published:** 2010-05-15

**Authors:** Shi Wang, Wenrui He, Xuehua Ding, Wei Huang

**Affiliations:** a Jiangsu Key Laboratory of Organic Electronics & Information Displays, Institute of Advanced Materials (IAM), Nanjing University of Posts and Telecommunications, Nanjing 210046, People’s Republic of China

## Abstract

In the title compound, [Cr_2_Ni(C_16_H_14_N_2_O_2_)_2_(CN)_4_(C_10_H_24_N_4_)]·2CH_3_OH, each [Cr(salen)(CN)_2_] unit {salen is 2,2′-[ethane-1,2-diylbis(nitrilo­methyl­idyne)]diphenolate} acts as a monodentate ligand through one of its two cyanide groups N bound to a central [Ni(cyclam)]^2+^ core (cyclam is 1,4,8,11-tetra­azacyclo­tetra­deca­ne). Each Cr^III^ ion is coordinated by two N and two O atoms from a salen ligand situated in the equatorial plane with two *trans* cyanide C atoms, yielding a distorted octa­hedral coordination geometry. The Ni^II^ atom lies on an inversion center and is octa­hedrally coordinated by a cyclam ligand lying in the equatorial plane and by two cyanide N atoms. The asymmetric unit contains one half of the complex mol­ecule and a methanol solvent mol­ecule. In the crystal structure, the complex mol­ecule is linked to the methanol solvent mol­ecules *via* O—H⋯O and N—H⋯O hydrogen bonds. Individual complex mol­ecules are linked by C—H⋯N hydrogen bonds, forming chains along *b*.

## Related literature

For general background to cyanide-bridged low-dimensional complexes and polynuclear clusters, see: Lescouëzec *et al.* (2005 ▶). For a related structure, see: Ni *et al.* (2008 ▶). For synthesis of the complex components, see: Yamada & Iwasaki (1969 ▶); Bosnich *et al.* (1965 ▶).
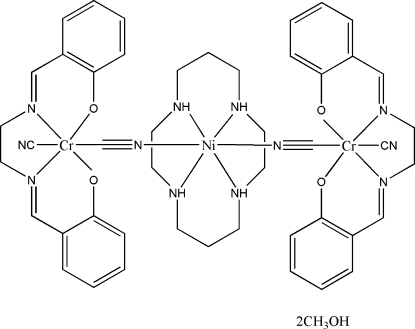


## Experimental

### 

#### Crystal data

[Cr_2_Ni(C_16_H_14_N_2_O_2_)_2_(CN)_4_(C_10_H_24_N_4_)]·2CH_4_O
*M* *_r_* = 1063.77
Monoclinic, 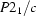

*a* = 9.5711 (19) Å
*b* = 18.936 (4) Å
*c* = 13.593 (3) Å
β = 103.93 (3)°
*V* = 2391.1 (9) Å^3^
*Z* = 2
Mo *K*α radiation
μ = 0.90 mm^−1^
*T* = 100 K
0.25 × 0.15 × 0.09 mm

#### Data collection

Bruker SMART CCD area-detector diffractometer
Absorption correction: multi-scan (*SADABS*; Sheldrick, 1996 ▶) *T*_min_ = 0.851, *T*_max_ = 0.922
5363 measured reflections
5363 independent reflections
5077 reflections with *I* > 2σ(*I*)

#### Refinement

*R*[*F* ^2^ > 2σ(*F* ^2^)] = 0.054
*wR*(*F* ^2^) = 0.132
*S* = 1.16
5363 reflections
314 parameters
H-atom parameters constrained
Δρ_max_ = 0.76 e Å^−3^
Δρ_min_ = −0.76 e Å^−3^

### 

Data collection: *SMART* (Bruker, 2007 ▶); cell refinement: *SAINT* (Bruker, 2007 ▶); data reduction: *SAINT*; program(s) used to solve structure: *SHELXS97* (Sheldrick, 2008 ▶); program(s) used to refine structure: *SHELXL97* (Sheldrick, 2008 ▶); molecular graphics: *SHELXTL* (Sheldrick, 2008 ▶); software used to prepare material for publication: *SHELXTL*.

## Supplementary Material

Crystal structure: contains datablocks I, global. DOI: 10.1107/S1600536810016995/sj2793sup1.cif

Structure factors: contains datablocks I. DOI: 10.1107/S1600536810016995/sj2793Isup2.hkl

Additional supplementary materials:  crystallographic information; 3D view; checkCIF report

## Figures and Tables

**Table 1:** Hydrogen-bond geometry (Å, °)

*D*—H⋯*A*	*D*—H	H⋯*A*	*D*⋯*A*	*D*—H⋯*A*
O3—H30⋯O2^i^	0.82	2.18	2.989 (3)	168
N5—H17⋯O3^ii^	0.91	2.34	3.212 (3)	161
C17—H12⋯N2^iii^	0.97	2.56	3.467 (4)	156

Symmetry codes: (i) 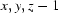; (ii) 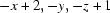; (iii) 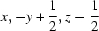.

**Fig. 1.**
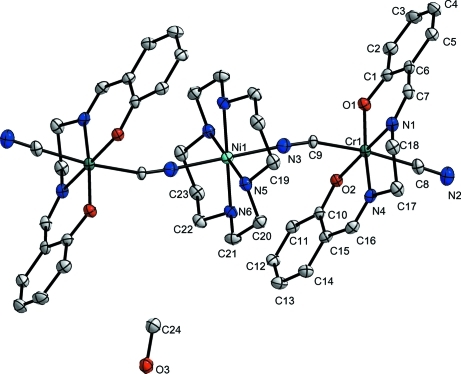
The molecular structure of (I), shown with 50% probability displacement ellipsoids.The unlabeled atoms are derived from the reference atoms by means of the (2-x, -y, 2-z) symmetry transformation.

**Fig. 2.**
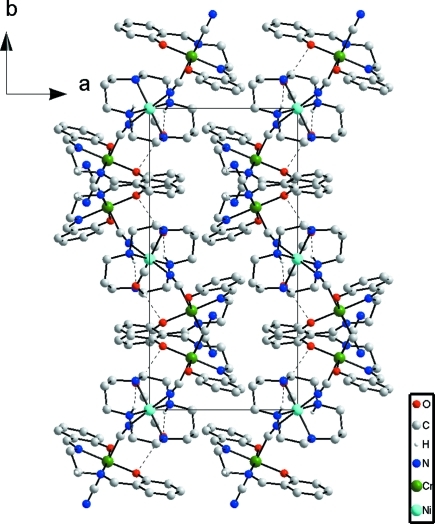
Packing diagram viewed down the c axis, The O—H···O and N—H···O hydrogen bonds are shown as dotted lines.

## supplementary crystallographic information

### Comment

Cyanide-bridged infinite systems (or Prussian blue analogues) and high-spin clusters have attracted great research interest due to their unique magnetic properties, including high-Tc superconducting magnets and photoinduced magnetization. Among these interesting researches, low-dimensional complexes as well as polynuclear clusters have attracted special attention, because they can be used to investigate the inter-metallic magnetic coupling quantitatively (Lescouëzec *et al.*, 2005).

Recently, a new cyanide-containing building block K[Cr(salen)(CN)_2_] (salen^2-^ = N,*N*'- bis(salicyl)ethylenediaminate) with two trans cyanide groups has been exploited to assemble cyanide-bridged low-dimensional complexes (Ni *et al.*, 2008). By using this new building block, we report here the synthesis and crystal structure of the title compound, [Cr(salen)(CN)_2_]_2_[Ni(cyclam)].CH_3_OH.

Complex I consists of a trinuclear cluster and one methanol solvate molecule. As shown in Fig. 1, in this trinuclear cluster, the [Cr(salen)(CN)_2_] unit acts as a monodentate ligand through one of its two cyanide groups toward a central [Ni(cyclam)]^2+^ core. The nickel atom is in an axially elongated octahedral environment.  Four nitrogen atoms from the cyclam ligand form the equatorial plane. Two cyanide nitrogen atoms occupy the axial positions. The complex are linked with the methanol solvate molecules *via* O—H···O and N—H···O hydrogen bonds (Fig. 2). The individual complex molecules are linked by C—H···N hydrogen bonds to form chains along *b*.

### Experimental

K[Cr(salen)(CN)_2_].H_2_O was synthesized according to the procedure described in the literature (Yamada *et al.*, 1969). Ni(cyclam)(ClO_4_)_2_ was synthesized as described previously (Bosnich *et al.*, (1965).

A solution of K[Cr(salen)(CN)_2_].H_2_O (79.6 mg, 0.2 mmol) in methanol (5 ml) was added dropwise to a solution of Ni(cyclam)(ClO_4_)_2_ (45.5 mg, 0.1 mmol) in water (3 ml). The mixture was stirred at room temperature for 5 min s and then filtered. Orange block crystals of (I) suitable for X-ray analysis were obtained by slow evaporation of the filtrate.

### Refinement

Aromatic H atoms were placed in calculated positions with C—H = 0.93 Å, and refined in riding mode with U_iso_(H) = 1.2U_eq_(C).

### Crystal data

**Table:**

[Cr_2_Ni(C_16_H_14_N_2_O_2_)_2_(CN)_4_(C_10_H_24_N_4_)]·2CH_4_O	*F*(000) = 1112
*M**_r_* = 1063.77	*D*_x_ = 1.477 Mg m^−^^3^
Monoclinic, *P*2_1_/*c*	Mo *K*α radiation, λ = 0.71073 Å
Hall symbol: -P 2ybc	Cell parameters from 7889 reflections
*a* = 9.5711 (19) Å	θ = 2.2–27.9°
*b* = 18.936 (4) Å	µ = 0.90 mm^−^^1^
*c* = 13.593 (3) Å	*T* = 100 K
β = 103.93 (3)°	Block, orange
*V* = 2391.1 (9) Å^3^	0.25 × 0.15 × 0.09 mm
*Z* = 2	

### Data collection

**Table:**

Bruker SMART CCD area-detector diffractometer	5363 independent reflections
Radiation source: fine-focus sealed tube	5077 reflections with *I* > 2σ(*I*)
graphite	*R*_int_ = 0.0000
Detector resolution: 8.366 pixels mm^-1^	θ_max_ = 27.5°, θ_min_ = 3.2°
φ and ω scans	*h* = 0→12
Absorption correction: multi-scan (*SADABS*; Sheldrick, 1996)	*k* = 0→24
*T*_min_ = 0.851, *T*_max_ = 0.922	*l* = −17→17
5363 measured reflections	

### Refinement

**Table:**

Refinement on *F*^2^	Primary atom site location: structure-invariant direct methods
Least-squares matrix: full	Secondary atom site location: difference Fourier map
*R*[*F*^2^ > 2σ(*F*^2^)] = 0.054	Hydrogen site location: inferred from neighbouring sites
*wR*(*F*^2^) = 0.132	H-atom parameters constrained
*S* = 1.16	*w* = 1/[σ^2^(*F*_o_^2^) + (0.0531*P*)^2^ + 5.7746*P*] where *P* = (*F*_o_^2^ + 2*F*_c_^2^)/3
5363 reflections	(Δ/σ)_max_ = 0.001
314 parameters	Δρ_max_ = 0.76 e Å^−^^3^
0 restraints	Δρ_min_ = −0.76 e Å^−^^3^

### Special details

**Table:**

Geometry. All esds (except the esd in the dihedral angle between two l.s. planes) are estimated using the full covariance matrix. The cell esds are taken into account individually in the estimation of esds in distances, angles and torsion angles; correlations between esds in cell parameters are only used when they are defined by crystal symmetry. An approximate (isotropic) treatment of cell esds is used for estimating esds involving l.s. planes.
Refinement. Refinement of *F*^2^ against ALL reflections. The weighted *R*-factor wR and goodness of fit *S* are based on *F*^2^, conventional *R*-factors *R* are based on *F*, with *F* set to zero for negative *F*^2^. The threshold expression of *F*^2^ > σ(*F*^2^) is used only for calculating *R*-factors(gt) etc. and is not relevant to the choice of reflections for refinement. *R*-factors based on *F*^2^ are statistically about twice as large as those based on *F*, and *R*- factors based on ALL data will be even larger.

### Fractional atomic coordinates and isotropic or equivalent isotropic displacement parameters (Å^2^)

**Table:**

	*x*	*y*	*z*	*U*_iso_*/*U*_eq_	
O1	1.2639 (2)	0.12757 (10)	1.40979 (14)	0.0198 (4)	
C1	1.3690 (3)	0.10694 (14)	1.4854 (2)	0.0196 (5)	
C2	1.3393 (3)	0.09542 (15)	1.5811 (2)	0.0238 (6)	
H1	1.2466	0.1031	1.5889	0.029*	
C3	1.4453 (4)	0.07288 (16)	1.6633 (2)	0.0292 (6)	
H2	1.4231	0.0671	1.7258	0.035*	
C4	1.5836 (4)	0.05875 (17)	1.6546 (2)	0.0302 (7)	
H3	1.6532	0.0424	1.7100	0.036*	
C5	1.6166 (3)	0.06930 (15)	1.5626 (2)	0.0249 (6)	
H4	1.7095	0.0600	1.5563	0.030*	
C6	1.5121 (3)	0.09400 (14)	1.4777 (2)	0.0205 (5)	
C7	1.5585 (3)	0.10267 (15)	1.3849 (2)	0.0211 (5)	
H5	1.6503	0.0871	1.3840	0.025*	
N1	1.4815 (2)	0.13043 (12)	1.30349 (17)	0.0181 (4)	
Cr1	1.28346 (4)	0.17173 (2)	1.28585 (3)	0.01505 (12)	
C8	1.3751 (3)	0.26188 (15)	1.3624 (2)	0.0211 (5)	
N2	1.4268 (3)	0.31239 (15)	1.4018 (2)	0.0315 (6)	
C9	1.1978 (3)	0.08685 (13)	1.19052 (19)	0.0150 (5)	
N3	1.1395 (3)	0.05280 (14)	1.1224 (2)	0.0262 (5)	
O2	1.08976 (19)	0.21092 (10)	1.25226 (14)	0.0174 (4)	
C10	1.0208 (3)	0.23457 (13)	1.1621 (2)	0.0171 (5)	
C11	0.8713 (3)	0.24589 (14)	1.1438 (2)	0.0201 (5)	
H6	0.8254	0.2372	1.1955	0.024*	
C12	0.7910 (3)	0.26948 (15)	1.0515 (2)	0.0230 (6)	
H7	0.6924	0.2760	1.0420	0.028*	
C13	0.8559 (3)	0.28365 (16)	0.9720 (2)	0.0263 (6)	
H8	0.8013	0.2989	0.9095	0.032*	
C14	1.0025 (3)	0.27454 (15)	0.9879 (2)	0.0226 (5)	
H9	1.0469	0.2852	0.9359	0.027*	
C15	1.0869 (3)	0.24956 (14)	1.0807 (2)	0.0183 (5)	
C16	1.2394 (3)	0.24080 (14)	1.0865 (2)	0.0205 (5)	
H10	1.2736	0.2560	1.0316	0.025*	
N4	1.3299 (2)	0.21365 (12)	1.16162 (17)	0.0179 (4)	
C17	1.4833 (3)	0.20758 (16)	1.1619 (2)	0.0231 (6)	
H11	1.5374	0.2463	1.1997	0.028*	
H12	1.4956	0.2089	1.0932	0.028*	
C18	1.5359 (3)	0.13719 (16)	1.2118 (2)	0.0222 (5)	
H13	1.5006	0.0986	1.1656	0.027*	
H14	1.6403	0.1358	1.2294	0.027*	
Ni1	1.0000	0.0000	1.0000	0.01755 (13)	
C19	1.2881 (3)	−0.00106 (16)	0.9439 (2)	0.0234 (6)	
H15	1.3293	0.0285	1.0019	0.028*	
H16	1.3413	0.0073	0.8926	0.028*	
N5	1.1361 (2)	0.01852 (13)	0.90325 (18)	0.0207 (5)	
H17	1.1026	−0.0078	0.8465	0.025*	
C20	1.1149 (3)	0.09359 (15)	0.8731 (2)	0.0240 (6)	
H18	1.1485	0.1018	0.8122	0.029*	
H19	1.1698	0.1235	0.9265	0.029*	
C21	0.9557 (3)	0.11151 (16)	0.8536 (2)	0.0250 (6)	
H20	0.9409	0.1611	0.8361	0.030*	
H21	0.9014	0.0837	0.7973	0.030*	
N6	0.9049 (3)	0.09605 (13)	0.94632 (17)	0.0211 (5)	
H22	0.9418	0.1298	0.9931	0.025*	
C22	0.7465 (3)	0.09755 (16)	0.9297 (2)	0.0239 (6)	
H23	0.7045	0.0646	0.8762	0.029*	
H24	0.7118	0.1444	0.9074	0.029*	
C23	0.6967 (3)	0.07874 (16)	1.0240 (2)	0.0251 (6)	
H25	0.5962	0.0919	1.0131	0.030*	
H26	0.7507	0.1071	1.0797	0.030*	
C24	0.9633 (4)	0.04217 (18)	0.3659 (3)	0.0322 (7)	
H27	1.0537	0.0235	0.3590	0.048*	
H28	0.8967	0.0041	0.3649	0.048*	
H29	0.9773	0.0672	0.4289	0.048*	
O3	0.9074 (2)	0.08903 (12)	0.28438 (17)	0.0290 (5)	
H30	0.9666	0.1201	0.2830	0.044*	

### Atomic displacement parameters (Å^2^)

**Table:**

	*U*^11^	*U*^22^	*U*^33^	*U*^12^	*U*^13^	*U*^23^
O1	0.0190 (9)	0.0243 (9)	0.0166 (9)	0.0000 (7)	0.0051 (7)	−0.0010 (7)
C1	0.0251 (13)	0.0169 (12)	0.0168 (12)	−0.0006 (10)	0.0047 (10)	−0.0012 (9)
C2	0.0291 (14)	0.0225 (13)	0.0217 (13)	−0.0005 (11)	0.0098 (11)	−0.0008 (10)
C3	0.0450 (18)	0.0252 (14)	0.0165 (13)	−0.0004 (13)	0.0057 (12)	0.0009 (11)
C4	0.0352 (16)	0.0289 (15)	0.0213 (14)	−0.0003 (12)	−0.0035 (12)	0.0014 (11)
C5	0.0224 (13)	0.0236 (13)	0.0252 (14)	−0.0003 (11)	−0.0009 (11)	0.0004 (11)
C6	0.0221 (13)	0.0190 (12)	0.0187 (12)	−0.0047 (10)	0.0018 (10)	−0.0033 (10)
C7	0.0165 (12)	0.0234 (13)	0.0233 (13)	0.0001 (10)	0.0047 (10)	−0.0046 (10)
N1	0.0161 (10)	0.0236 (11)	0.0163 (10)	−0.0017 (8)	0.0073 (8)	−0.0038 (8)
Cr1	0.0139 (2)	0.0187 (2)	0.0133 (2)	−0.00180 (14)	0.00477 (15)	−0.00241 (14)
C8	0.0179 (12)	0.0277 (14)	0.0182 (12)	−0.0023 (10)	0.0055 (10)	−0.0047 (10)
N2	0.0283 (13)	0.0338 (14)	0.0339 (14)	−0.0063 (11)	0.0102 (11)	−0.0067 (11)
C9	0.0132 (10)	0.0153 (11)	0.0165 (11)	−0.0002 (9)	0.0035 (9)	−0.0035 (9)
N3	0.0231 (12)	0.0284 (13)	0.0282 (13)	0.0007 (10)	0.0083 (10)	0.0018 (10)
O2	0.0157 (8)	0.0225 (9)	0.0151 (9)	−0.0011 (7)	0.0056 (7)	0.0000 (7)
C10	0.0179 (12)	0.0165 (11)	0.0167 (12)	−0.0006 (9)	0.0038 (9)	−0.0032 (9)
C11	0.0189 (12)	0.0195 (12)	0.0239 (13)	0.0014 (10)	0.0091 (10)	0.0020 (10)
C12	0.0171 (12)	0.0222 (13)	0.0295 (15)	0.0033 (10)	0.0049 (11)	0.0042 (11)
C13	0.0261 (14)	0.0271 (14)	0.0253 (14)	0.0058 (11)	0.0057 (11)	0.0074 (11)
C14	0.0241 (13)	0.0227 (13)	0.0219 (13)	0.0020 (10)	0.0071 (11)	0.0031 (10)
C15	0.0194 (12)	0.0178 (12)	0.0192 (12)	0.0003 (9)	0.0077 (10)	0.0005 (9)
C16	0.0234 (13)	0.0211 (12)	0.0203 (13)	−0.0021 (10)	0.0121 (11)	0.0000 (10)
N4	0.0155 (10)	0.0221 (11)	0.0188 (11)	−0.0031 (8)	0.0092 (8)	−0.0009 (8)
C17	0.0155 (12)	0.0356 (15)	0.0204 (13)	−0.0018 (11)	0.0084 (10)	0.0014 (11)
C18	0.0185 (12)	0.0295 (14)	0.0205 (13)	0.0013 (10)	0.0082 (10)	−0.0025 (11)
Ni1	0.0168 (2)	0.0196 (2)	0.0167 (2)	−0.00043 (17)	0.00499 (18)	−0.00011 (17)
C19	0.0179 (13)	0.0315 (15)	0.0223 (14)	0.0001 (10)	0.0077 (11)	−0.0021 (11)
N5	0.0202 (11)	0.0236 (11)	0.0187 (11)	−0.0001 (9)	0.0053 (9)	−0.0027 (9)
C20	0.0256 (13)	0.0237 (13)	0.0241 (13)	−0.0018 (11)	0.0089 (11)	0.0020 (11)
C21	0.0316 (15)	0.0248 (14)	0.0198 (13)	0.0036 (11)	0.0087 (11)	0.0025 (10)
N6	0.0221 (11)	0.0230 (11)	0.0176 (11)	0.0009 (9)	0.0038 (9)	−0.0010 (9)
C22	0.0224 (13)	0.0257 (14)	0.0227 (13)	0.0042 (11)	0.0041 (11)	−0.0007 (11)
C23	0.0218 (13)	0.0303 (15)	0.0235 (14)	0.0040 (11)	0.0060 (11)	−0.0025 (11)
C24	0.0296 (15)	0.0342 (17)	0.0319 (16)	−0.0043 (13)	0.0059 (13)	0.0031 (13)
O3	0.0240 (10)	0.0339 (11)	0.0288 (11)	−0.0053 (9)	0.0058 (9)	0.0000 (9)

### Geometric parameters (Å, °)

**Table:**

O1—C1	1.313 (3)	C16—H10	0.9300
O1—Cr1	1.930 (2)	N4—C17	1.472 (3)
C1—C2	1.413 (4)	C17—C18	1.525 (4)
C1—C6	1.420 (4)	C17—H11	0.9700
C2—C3	1.384 (4)	C17—H12	0.9700
C2—H1	0.9300	C18—H13	0.9700
C3—C4	1.383 (5)	C18—H14	0.9700
C3—H2	0.9300	Ni1—N6^i^	2.086 (2)
C4—C5	1.376 (5)	Ni1—N6	2.086 (2)
C4—H3	0.9300	Ni1—N5^i^	2.092 (2)
C5—C6	1.413 (4)	Ni1—N5	2.092 (2)
C5—H4	0.9300	Ni1—N3^i^	2.117 (3)
C6—C7	1.443 (4)	C19—N5	1.473 (4)
C7—N1	1.285 (4)	C19—C23^i^	1.531 (4)
C7—H5	0.9300	C19—H15	0.9700
N1—C18	1.467 (3)	C19—H16	0.9700
N1—Cr1	2.011 (2)	N5—C20	1.480 (4)
Cr1—O2	1.9464 (19)	N5—H17	0.9100
Cr1—N4	2.010 (2)	C20—C21	1.521 (4)
Cr1—C8	2.080 (3)	C20—H18	0.9700
Cr1—C9	2.103 (2)	C20—H19	0.9700
C8—N2	1.148 (4)	C21—N6	1.485 (4)
C9—N3	1.156 (4)	C21—H20	0.9700
N3—Ni1	2.117 (3)	C21—H21	0.9700
O2—C10	1.322 (3)	N6—C22	1.478 (4)
C10—C11	1.408 (4)	N6—H22	0.9100
C10—C15	1.429 (4)	C22—C23	1.513 (4)
C11—C12	1.379 (4)	C22—H23	0.9700
C11—H6	0.9300	C22—H24	0.9700
C12—C13	1.396 (4)	C23—C19^i^	1.531 (4)
C12—H7	0.9300	C23—H25	0.9700
C13—C14	1.378 (4)	C23—H26	0.9700
C13—H8	0.9300	C24—O3	1.420 (4)
C14—C15	1.406 (4)	C24—H27	0.9600
C14—H9	0.9300	C24—H28	0.9600
C15—C16	1.453 (4)	C24—H29	0.9600
C16—N4	1.277 (4)	O3—H30	0.8200
			
C1—O1—Cr1	126.52 (18)	C18—C17—H12	110.3
O1—C1—C2	118.7 (3)	H11—C17—H12	108.6
O1—C1—C6	124.3 (2)	N1—C18—C17	107.9 (2)
C2—C1—C6	117.0 (3)	N1—C18—H13	110.1
C3—C2—C1	121.3 (3)	C17—C18—H13	110.1
C3—C2—H1	119.4	N1—C18—H14	110.1
C1—C2—H1	119.4	C17—C18—H14	110.1
C4—C3—C2	121.4 (3)	H13—C18—H14	108.4
C4—C3—H2	119.3	N6^i^—Ni1—N6	180.000 (1)
C2—C3—H2	119.3	N6^i^—Ni1—N5^i^	85.37 (9)
C5—C4—C3	118.9 (3)	N6—Ni1—N5^i^	94.63 (9)
C5—C4—H3	120.6	N6^i^—Ni1—N5	94.63 (9)
C3—C4—H3	120.6	N6—Ni1—N5	85.37 (9)
C4—C5—C6	121.2 (3)	N5^i^—Ni1—N5	180.0
C4—C5—H4	119.4	N6^i^—Ni1—N3^i^	90.15 (10)
C6—C5—H4	119.4	N6—Ni1—N3^i^	89.85 (10)
C5—C6—C1	120.1 (3)	N5^i^—Ni1—N3^i^	92.54 (10)
C5—C6—C7	116.4 (3)	N5—Ni1—N3^i^	87.46 (9)
C1—C6—C7	123.4 (2)	N6^i^—Ni1—N3	89.85 (10)
N1—C7—C6	124.4 (3)	N6—Ni1—N3	90.15 (10)
N1—C7—H5	117.8	N5^i^—Ni1—N3	87.46 (9)
C6—C7—H5	117.8	N5—Ni1—N3	92.54 (10)
C7—N1—C18	121.3 (2)	N3^i^—Ni1—N3	180.0
C7—N1—Cr1	126.18 (19)	N5—C19—C23^i^	111.5 (2)
C18—N1—Cr1	112.47 (17)	N5—C19—H15	109.3
O1—Cr1—O2	94.74 (8)	C23^i^—C19—H15	109.3
O1—Cr1—N4	172.76 (9)	N5—C19—H16	109.3
O2—Cr1—N4	92.47 (9)	C23^i^—C19—H16	109.3
O1—Cr1—N1	90.82 (9)	H15—C19—H16	108.0
O2—Cr1—N1	173.49 (9)	C19—N5—C20	113.8 (2)
N4—Cr1—N1	82.02 (10)	C19—N5—Ni1	115.47 (18)
O1—Cr1—C8	92.10 (10)	C20—N5—Ni1	105.70 (17)
O2—Cr1—C8	93.88 (10)	C19—N5—H17	107.2
N4—Cr1—C8	86.81 (10)	C20—N5—H17	107.2
N1—Cr1—C8	89.29 (10)	Ni1—N5—H17	107.2
O1—Cr1—C9	95.82 (9)	N5—C20—C21	109.1 (2)
O2—Cr1—C9	86.51 (9)	N5—C20—H18	109.9
N4—Cr1—C9	85.20 (10)	C21—C20—H18	109.9
N1—Cr1—C9	89.57 (9)	N5—C20—H19	109.9
C8—Cr1—C9	172.01 (11)	C21—C20—H19	109.9
N2—C8—Cr1	177.7 (3)	H18—C20—H19	108.3
N3—C9—Cr1	164.0 (2)	N6—C21—C20	109.1 (2)
C9—N3—Ni1	170.0 (2)	N6—C21—H20	109.9
C10—O2—Cr1	125.54 (16)	C20—C21—H20	109.9
O2—C10—C11	118.3 (2)	N6—C21—H21	109.9
O2—C10—C15	124.7 (2)	C20—C21—H21	109.9
C11—C10—C15	117.0 (2)	H20—C21—H21	108.3
C12—C11—C10	122.0 (3)	C22—N6—C21	113.6 (2)
C12—C11—H6	119.0	C22—N6—Ni1	114.53 (18)
C10—C11—H6	119.0	C21—N6—Ni1	105.33 (17)
C11—C12—C13	120.8 (3)	C22—N6—H22	107.7
C11—C12—H7	119.6	C21—N6—H22	107.7
C13—C12—H7	119.6	Ni1—N6—H22	107.7
C14—C13—C12	118.7 (3)	N6—C22—C23	112.8 (2)
C14—C13—H8	120.6	N6—C22—H23	109.0
C12—C13—H8	120.6	C23—C22—H23	109.0
C13—C14—C15	121.7 (3)	N6—C22—H24	109.0
C13—C14—H9	119.1	C23—C22—H24	109.0
C15—C14—H9	119.1	H23—C22—H24	107.8
C14—C15—C10	119.7 (2)	C22—C23—C19^i^	116.1 (2)
C14—C15—C16	116.1 (2)	C22—C23—H25	108.3
C10—C15—C16	124.2 (2)	C19^i^—C23—H25	108.3
N4—C16—C15	124.6 (2)	C22—C23—H26	108.3
N4—C16—H10	117.7	C19^i^—C23—H26	108.3
C15—C16—H10	117.7	H25—C23—H26	107.4
C16—N4—C17	121.2 (2)	O3—C24—H27	109.5
C16—N4—Cr1	125.90 (19)	O3—C24—H28	109.5
C17—N4—Cr1	112.89 (17)	H27—C24—H28	109.5
N4—C17—C18	107.0 (2)	O3—C24—H29	109.5
N4—C17—H11	110.3	H27—C24—H29	109.5
C18—C17—H11	110.3	H28—C24—H29	109.5
N4—C17—H12	110.3	C24—O3—H30	109.5

Symmetry codes: (i) −*x*+2, −*y*, −*z*+2.

### Hydrogen-bond geometry (Å, °)

**Table:**

*D*—H···*A*	*D*—H	H···*A*	*D*···*A*	*D*—H···*A*
O3—H30···O2^ii^	0.82	2.18	2.989 (3)	168
N5—H17···O3^iii^	0.91	2.34	3.212 (3)	161
C17—H12···N2^iv^	0.97	2.56	3.467 (4)	156

Symmetry codes: (ii) *x*, *y*, *z*−1; (iii) −*x*+2, −*y*, −*z*+1; (iv) *x*, −*y*+1/2, *z*−1/2.
